# Pyroptosis: A New Frontier in Kidney Diseases

**DOI:** 10.1155/2021/6686617

**Published:** 2021-04-28

**Authors:** Ke-jia Zhang, Qi Wu, Shi-min Jiang, Lei Ding, Chao-xia Liu, Ming Xu, Ying Wang, Yao Zhou, Li Li

**Affiliations:** ^1^Department of Pathophysiology, Xuzhou Medical University, Xuzhou 221009, China; ^2^Laboratory of Clinical and Experimental Pathology, Xuzhou Medical University, Xuzhou 221009, China; ^3^Department of Physiology, Xuzhou Medical University, Xuzhou 221009, China

## Abstract

Pyroptosis is a pattern of programmed cell death that significantly differs from apoptosis and autophagy in terms of cell morphology and function. The process of pyroptosis is characterized predominantly by the formation of gasdermin protein family-mediated membrane perforation, cell collapse, and the release of inflammatory factors, including IL-1*β* and IL-18. In recent years, with the rise of pyroptosis research, scholars have devoted time to study the mechanism of pyroptosis in kidney-related diseases. Pyroptosis is probably involved in kidney diseases through two pathways: the caspase-1-mediated canonical pathway and the caspase-4/5/11-mediated noncanonical pathway. In addition, some scholars have identified targets for the treatment of kidney-related diseases from the viewpoint of pyroptosis and developed corresponding medicines, which may become a recommendation for prognosis, targeted treatment, and clinical diagnosis of kidney diseases. This paper focuses on the up-to-date advances in the field of pyroptosis, especially on the key pathogenic role of pyroptosis in the development and progression of kidney diseases. It presents a more in-depth understanding of the pathogenesis of kidney diseases and introduces novel therapeutic targets for the prevention and clinical treatment of kidney diseases.

## 1. Introduction

Kidney diseases are a global health problem, affecting more than 750 million people worldwide; they are diseases with a high incidence and mortality. Representative kidney diseases include acute kidney injury (AKI) and chronic kidney disease (CKD). It is estimated that AKI leads to approximately 1.7 million deaths every year worldwide, and the incidence of CKD worldwide is approximately 11–13% [1]. The prevalence of risk factors for kidney disease continues to increase. However, given the large variation between countries in social economy, culture, and politics, there are many differences in examination methods and assessment plans, leading to a poor curative efficiency and high healthcare burden [2, 3].

Programmed cell death plays a very important role in the maintenance of cellular homeostasis [4]. Pyroptosis is a type of proinflammatory programmed cell death; it is activated by an inflammation-related caspase family member, which cleaves gasdermin to expose its NT terminal, translocates to the membrane, and perforates the membrane, modifying intracellular osmotic pressure and finally leading to cell collapse [5]. Pyroptosis mainly occurs in myeloid-derived phagocytes, such as macrophages, dendritic cells, and neutrophilic granulocytes. Recent studies have indicated that pyroptosis is also observed in CD4^+^ T cells, keratinocytes, epithelial cells, and neurons [6]. Pyroptosis is mainly regulated by the caspase-1-mediated canonical pathway and caspase-4/5/11-mediated noncanonical pathway.

Subsequent in-depth studies revealed that pyroptosis is involved in the occurrence and development of many diseases, particularly kidney diseases. This review highlights the advances made in research into the relationship between pyroptosis and kidney diseases and discusses the potential therapeutic targets relating to the mechanism of pyroptosis in kidney diseases.

## 2. Pyroptosis

Brennan and Cookson found that *Salmonella typhi* could induce macrophage death via a caspase-1-dependent mechanism, which was different from previous knowledge about apoptosis [7, 8]. This type of cell death is known as pyroptosis. Pyroptosis differs from apoptosis and necrosis in terms of morphology and mechanism (Table 1). During pyroptosis, the cell membrane becomes damaged and a 1.1–2.4 nm perforation is formed; due to changes in intracellular and extracellular osmotic pressure, cells become tumid and cracked, membrane integrity is lost, and inflammatory factors are released. However, during this process, the structure and function of mitochondria remain intact [9, 10].

## 3. Signaling Pathway Related to Pyroptosis

**Figure 1:**
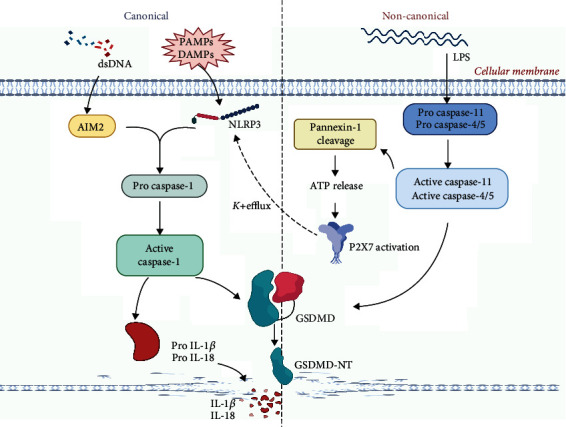
Canonical caspase-1-dependent and noncanonical caspase-4/5/11-mediated pyroptosis pathways [20, 33, 36, 37, 39, 43, 44, 48, 49]. The canonical pyroptosis signaling pathway: the NLRP3 inflammatory body is activated by pathogen- associated molecular pattern (PAMP) or damage-associated molecular pattern (DAMP), and the AIM2 inflammatory body is activated by dsDNA. They induce the activation of downstream caspase-1. On one hand, this promotes the release of inflammatory factors IL-1*β* and IL-18; on the other hand, this specifically cleaves GSDMD. The GSDMD-N terminal fragment aggregates at the cell membrane to cause membrane perforation and induce pyroptosis. Noncanonical pyroptosis signaling pathway: lipopolysaccharide (LPS) directly activates caspase-11 (human homology caspase-4/5) then cleaves GSDMD to form the GSDMD-N terminal, inducing pyroptosis. Pannexin-1 is cleaved and lysed by the activated caspase-11 (homologous to human caspase-4/5), which damages the channel for membrane small molecule release, leads to leakage of intracellular ATP, activates the ligand-gated channels (P2X7) of purinergic receptor P2X, accelerates K^+^ efflux, mediates the NLRP3/caspase-1 signaling pathway, and indirectly promotes the release of inflammatory factors IL-1*β* and IL-18.

The accumulated evidence has identified two pathways related to pyroptosis (Figure 1): the caspase-1-mediated canonical pathway that is induced by inflammatory bodies and the caspase-4/5/11-mediated noncanonical pathway that is activated by lipopolysaccharide (LPS).

### 3.1. Canonical Pyroptosis Pathway

The canonical pyroptosis pathway is mediated by inflammatory bodies, mainly nucleotide-binding oligomerization domain-like (NOD) receptor NLR family and the PYHIN protein family [15]. The inflammatory bodies can be activated by pathogen-associated molecular patterns (PAMPs) or damage-associated molecular patterns (DAMPs). The activation of caspase-1 by such inflammatory bodies may lead to pyroptosis.

The NLR protein family is composed mainly of a nucleotide-binding oligomerization domain (NOD/NACHT), a C-terminal containing leucine-rich repeats (LRRs), and an N-terminal recruiting caspase domain (CARD) or Pyrin domain (PYD) [16]. The NOD/NACHT domain is shared by the NLR family, and its downstream signal is activated by ATP-dependent oligomerization. The main functions of LRRs are related to ligand sense and autoregulation, whereas the CARD and PYD domains are the main mediators of the interaction between similar proteins in the downstream signals [17, 18].

Frequently observed inflammatory bodies in the NLR family include NLRP3, NLRP1, NLRP6, and NLRP6; they induce pyroptosis through the recruitment of caspase-1 via the adaptor protein of CARD-PYRIN [19]. Much attention has been paid to the inflammatory body NLRP3; as an intracellular sensor, it can be activated by a majority of endogenous risk factors and environmental stimulants [20]. Currently, it is known that NLRP3 can be activated in two ways: one is dependent on microbial molecules or endogenous cytokines; the other is induced by APT, pore-forming toxins, viral RNA, and particulates [21]. A further study revealed that the activation of NLRP3 could lead to the release of caspase-1-mediated proinflammatory cytokines, such as IL-1*β* and IL-18, and induce pyroptosis [20]. It was reported that anthrax lethal toxin (LT) could activate caspase-1-mediated macrophage pyroptosis, whereas caspase-1 was activated by LT-mediated cleavage of the 10 loci of NLPR1 and activation of inflammatory bodies [22]. Consequently, activation of the NLRP1 inflammatory body plays a very important role in the release of IL-induced inflammatory factors, such as IL-1*β* and IL-18, and pyroptosis [23, 24]. The NLRP6 inflammatory body is usually activated by microbiota-associated metabolites such as taurine or *Porphyromonas gingivalis*. It can also regulate the caspase-1-mediated pyroptosis of intestinal epithelial cells or human gingival fibroblasts [25, 26]. In 2017, Zhu et al. [27] found that NLRP9 could recognize a short double-stranded RNA extension through the RNA helicase Dhx9 and form an inflammatory complex with the adaptor proteins ASC and caspase-1, thereby promoting the release of IL-1*β* and IL-18 and inducing pyroptosis.

The pyroptosis mentioned above deals with the interaction between the adaptor protein ASC and caspase-1. Therefore, it has been examined whether the adaptor protein ASC is necessary during the entire process of inflammatory body-mediated pyroptosis. A previous study of macrophages has shown that when NAIP1/2 and NAIP5/6, two members of the NLR apoptosis-inhibition protein (NAIP) family, were activated by type III secretion system (T3SS) and flagellin, they can form the NLRC4-NAIP1/2 complex, or the NLRC4-NAIP5/6 complex, and recruit caspase-1 through the CARD-CARD interaction to trigger pyroptosis [28–30]. ASC does not participate in this process. Instead, NLRC4 directly recruits caspase-1 to trigger pyroptosis. This is probably because NLRC4 contains the CARD domain [31].

In addition to the NLR family, other inflammatory bodies also participate in the occurrence and development of pyroptosis. For example, cytoplasmic dsDNA and the dsDNA vaccinia virus could bind to the HIN200 domain in the absence of melanoma 2 (AIM2), leading to the activation of the PYD of AIM2 and the formation of a protein complex with AIM2, ASC, and caspase-1 and ultimately inducing pyroptosis [32, 33]. The mechanism for the regulation of pyroptosis by pyrin, the protein encoded by MEFV, is similar to that of AIM2. The human pyrin protein has four functional domains, the pyrin domain (PYD), a zinc finger domain (bBox), a coiled coil domain (CC), and a B30.2/SPRY domain. Mouse pyrin has three domains: PYD, bBox, and CC. As a pattern recognition receptor, pyrin does not directly recognize the risk factors of pathogens or hosts. Instead, the inactivation of RhoA GTPase induced by pathogens can activate the interaction between pyrin and caspase-1 and PYD, which finally induces caspase-1-mediated immune pyroptosis [34].

The direct or indirect recruitment of caspase-1 by inflammatory bodies to regulate pyroptosis has been well studied. However, the mechanism through which activated caspase-1 mediates pyroptosis remains unknown. In 2015, Shao et al. found, in an *in vitro* experiment, that when caspase-1 was activated in mouse marrow macrophages, it could specifically cleave the gasdermin-N and gasdermin-C fragments from gasdermin (GSDMD) [35]. It was further confirmed that the GSDMD-N fragment could induce extensive cell death, which is much closer to pyroptosis. *In vitro* tests suggested that GSDMD-NT formation and the activation of caspase-1 probably occurred simultaneously and that ASC was not necessary for the activation of GSDMD. This implies that cleaved caspase-1 can not only cleave GSDMD but also directly activate GSDMD [36]. When GSDMD is cleaved into a mass of GSDMD-NT, such fragments migrate to the membrane while aggregating and perforating the membrane, leading to the release of inflammatory factors, such as IL-1*β* and IL-18. As the number of holes increases, the membrane breaks and the cellular contents such as IL-1*α* and high-mobility group box 1 protein (HMGB1) are discharged, causing the characteristic changes of pyroptosis [37, 38].

### 3.2. Noncanonical Pyroptosis Signaling Pathway

In addition to the canonical pyroptosis signaling pathway, the noncanonical pyroptosis signaling pathway has also been well studied. In 2011, pyroptosis in macrophages infected with *E. coli*, *Citrobacter corynii*, or *Vibrio cholera* is not dependent on inflammatory bodies, but caspase-11 [39]. Lipopolysaccharide (LPS) is the main component of the external membrane of gram-negative bacteria, and extracellular LPS can be recognized by Toll-like receptor 4 (TLR-4) to stimulate the transcription of cytokines. It was found that LPS in macrophages could activate caspase-11 and, during the activation, cholera toxin B is the cytoplasmic carrier of LPS and that LPS could activate caspase-11 independently of TLR4, suggesting that caspase-11 directly responds to cytoplasmic LPS [40–42]. It was also found that caspase-11 and homologous to human caspase-4 and caspase-5 could directly bind to LPS to self-activate, thereby inducing pyroptosis, although the role of LPS in the activation of caspase-11 and human caspase homology requires further investigation [43]. It was also found that GSDMD was necessary for pyroptosis in caspase-4/5-mediated mouse macrophages [44]. Similarly, Aglietti et al. [45] reported that p30 protein fragments derived from GSDMD cleavage after caspase-11 activation could bind to the membrane, leading to the characteristic morphology of pyroptosis and membrane perforation. An up-to-date study found that the Asp289/285 loci of caspase-4/11 were crucial for the induction of pyroptosis, which again expanded our knowledge of pyroptosis [46].

Therefore, it should be considered whether caspase-11-mediated pyroptosis was related to caspase-1. Pannexin-1 is a membrane channel protein expressed extensively in all kinds of tissues and cells and can be cleaved and lysed by caspase-11, damaging the channel for membrane small molecule release, leading to the efflux of intracellular ATP, activating the ligand-gated ion channels (P2X7) of purinergic receptor P2X, and mediating the pyroptosis of macrophages [47]. It was also found that exogenous APT could activate P2X7 to form a channel for K^+^ efflux, which is crucial for the activation of the NLRP3 inflammatory body. Therefore, it is likely that the caspase-11-mediated noncanonical inflammation pathway could promote K^+^ efflux via pannexin-1, thereby activating the NLRP3 inflammatory body and caspase-1 and eventually leading to pyroptosis, maturation, and the release of inflammatory factors [48, 49].

Several studies have shown that caspase-8 plays an important role in the pathogen-induced inflammatory response of macrophages. In 2018, it was found that activated caspase-8 could lead to cell membrane damage and K^+^ outflow, which further activates the NLRP3 inflammasome and triggers oligomerization of ASC. As a result, IL-1*β* is released, and during this process, GSDMD plays a central role [50]. In the same year, another group also found that caspase-8 could lead to the lysis of GSDMD and GSDME in murine macrophages, triggering IL-1*β* release and pyroptosis. Moreover, in a mouse model of colitis, the inflammatory response was also found to be closely related to caspase-8-mediated pyroptosis [51]. Therefore, it is proposed that caspase-8 is a switch for pyroptosis [52]. However, it remains unclear whether caspase-8 split-cleaves GSDMD directly or whether any intermediates are required. Until 2020, it was believed that caspase-8 functions through the direct cleavage of GSDMD and that the activity of caspase-8 on different complexes correlates to its capacity to directly cleave GSDMD [53, 54]. At present, studies regarding the correlation between caspase-8 and pyroptosis are still emerging, and many related scientific concepts require further investigation.

In addition, activated caspase-3 could also lyse GSDME to induce pyroptosis [55]. In 2019, Nomenclature Committee on Cell Death (NCCD) modified the definition of pyroptosis and defined it as a cell death pattern dependent on the gasdermin family protein involved in membrane perforation. However, pyroptosis is not always dependent on the induction of inflammatory caspases. In addition, the type of cell death is not determined by the type of caspase, but by the substrates cleaved by caspase [5].

## 4. Association between Pyroptosis and Kidney Disease

Common kidney diseases that may lead to end-stage renal disease (ESRD) include acute kidney injury, diabetic kidney disease, renal fibrosis, and kidney inflammation. It has been confirmed that all types of kidney disease are induced by a certain level of inflammatory reactions. Pyroptosis, regulated by a variety of inflammatory bodies, plays a very important role in the progression of kidney disease. In addition, there are various modulatory mechanisms of kidney diseases.

### 4.1. Acute Kidney Injury

Acute kidney injury (AKI) is induced by multiple factors that are characterized by a rapid decline in kidney function and the accumulation of metabolic wastes and toxins; eventually, complications and the failure of other organs may occur. The main pathogenic traits of AKI are renal tubular epithelial cell injury, interstitial inflammation, and the dysfunction of blood vessels. Ischemic reperfusion, endogenous, and exogenous nephrotoxins, including contrast medium, are common factors of AKI [56]. Numerous studies have indicated that these factors mentioned that induce AKI are associated with pyroptosis.

In 2010, Shigeoka et al. [57] reported that NLRP3 was highly expressed in renal tubular epithelial cells of mice and humans but was significantly enhanced in kidney IRI, suggesting the crucial role of NLRP3 in IRI in the kidney. Yang et al. [58] was the first group to report that pyroptosis was a key event during IRI of the kidney; the overactivation of the endoplasmic reticulum stress-triggered CHOP-caspase-11 signaling pathway plays a crucial role in the pyroptosis of renal tubular epithelial cells after kidney IRI. This will likely support further studies of the relationship between pyroptosis and acute kidney injury. AKI is previously known as acute kidney failure, and the study found that repurified LPS can activate the caspase-1/IL-1*β* pathway during acute kidney failure, leading to pyroptosis [59].

In 2019, the expression of caspase-11 was shown to be markedly increased in tubular cell models of cis-platinum-induced AKI. The activation of caspase-11 cleaves GSDMD into GSDMD-NT, which was then translocated to the plasma membrane, triggering pyroptosis and promoting the release of IL-18 [60]. Iodized contrast agents can also lead to kidney disease. It is likely that the contrast agents damage the membrane of epithelial cells, entering into the cells to activate caspase-4/5/11, to cleave GSDMD, and to initiate pyroptosis [61]. An up-to-date study found that pyroptosis mediated by the caspase-11/GSDMD signaling pathway not only affected the release of cytokines but also determined the severity of AKI following septic shock [62]. These results suggest a close correlation between the pathogenesis of AKI and the caspase-4/5/11-mediated atypical pathway in pyroptosis. In addition, a recent study found that caspase-3/GSDME-mediated pyroptosis is also related to the occurrence and development of AKI. Genetic intervention and pharmacological studies with GSDME-deficient mice and human renal tubular epithelial cells showed that caspase-3 could alleviate pyroptosis and delay AKI by blocking GSDME lysis [63]. Therefore, targeted pyroptosis may be a novel approach for the treatment of AKI.

### 4.2. Diabetic Kidney Disease

Diabetic kidney disease (DKD) is a microvascular complication of diabetes. This is a type of disease caused by dysfunctional sugar metabolism. DKD is characterized by glomerular scarring, urine protein, and reduced renal function. The main histological property of DKD is the thickness of the glomerular basement membrane, dilation of glomerular mesangial, loss of Sertoli cells, fibrosis of glomeruli, and progressive fibrosis. Currently, DKD is mainly treated with hypoglycemia and better blood glucose control. However, this does not lead to a reduction in DKD. Therefore, it is important to investigate the pathogenesis of DKD [63].

In 2011, the NLRP3-ASC-caspase-1 signaling pathway was found to promote diabetes by regulating the release of IL-1*β* and other inflammatory factors [64]. It was also found that continuous hyperglycemia could activate the NLRP3 inflammatory body, promote the release of IL-1*β* and IL-18, and eventually lead to the occurrence and progression of DKD [65]. Knockout of NLRP3 gene in mice delayed DKD and alleviated kidney injury through inhibition of inflammatory reactions [66]. Serum NLRP3 mRNA is also a biomarker used to identify patients with DN [67]. This is indicative of the important role of NLRP3 in regulating the occurrence and progression of DKD.

The NLRP3 inflammatory body is one of the most common targets for the regulation of pyroptosis. Therefore, we considered whether pyroptosis participated in the occurrence and development of DKD. The results of the in vitro study showed that the expression of pyroptosis-related proteins, such as cleaved caspase-1, GSDMD, and the N terminal of GSDMD (GSDMD-NT), was enhanced during the progression of DKD, and that a marked release of inflammatory factors was also observed [68, 69].

In recent years, the close correlation among long noncoding RNAs (lncRNAs), pyroptosis, and DN has received much attention. It has been shown that RNA MALAT1 can inhibit miR-23c expression and promote hyperglycemia-induced pyroptosis of renal tubular epithelial cells by inhibiting miR-30c via the activation of NLRP3 [70]. A recent study showed that NEAT1/miR-34c/NLRP3-modulated pyroptosis and the subsequent inflammation could promote the progression of DKD [71]. In summary, the expression of lncRNAs is positively correlated with pyroptosis and is a potential biomarker for DKD. At present, the majority of studies concerning DKD are concentrated on the caspase-1-mediated typical pyroptosis pathway, which may provide a better understanding and a more effective method for the prevention and treatment of DKD.

### 4.3. Renal Fibrosis

Wound, infection, inflammation, blood circulatory disorder, immune response, and many other pathogenic factors can enhance fibroblasts and massive accumulation of collagen in the renal interstitium, leading to renal interstitial fibrosis and eventually ESRD.

Increased expression of NLRP3 and caspase-1, as well as enhanced release of the IL-1*β* and IL-18 inflammatory bodies, was observed in both renal biopsy specimens of UUO mice and humans with UUO (unilateral ureteral occlusion), whereas the degree of renal fibrosis was markedly alleviated in NLRP3^−/−^ mice [72, 73]. In a long-term study, Miao et al. [74] found in 2018 that in UUO mice caspase-11 could activate caspases-1, enhance the release of inflammatory factors, and promote the progression of renal fibrosis; pyroptosis is a UUO mediated by the atypical caspase-11 pathway. Necrotic DNA can exacerbate UUO-induced kidney disease and injury by activating the inflammatory body AIM2 [75]. The relationship between pyroptosis and renal fibrosis has been well studied. In 2012, pyroptosis was found to participate in renal interstitial fibrosis in a mouse model of UUO; this participation is probably related to inflammation, but the mechanism remains unknown [76]. In 2016, Xu et al. [77] found that the loss of MAP1S led to an accumulation of mouse renal fibrosis-related proteins and renal fibrosis in older mice. In addition, the inhibition of MAP1S in near-end renal tubular cells may lead to the development or progression of pyroptosis, which is further suggestive of the close relationship between pyroptosis and renal fibrosis. In addition, a recent study showed that caspase-3/GSDME-mediated UUO is related to renal tubular pyroptosis in mice. This suggests a role for intraureteral obstruction in renal tubular injury, resulting in kidney fibrosis [78]. However, only a few studies have addressed their correlation and a more in-depth study is required in the future.

### 4.4. Inflammatory Kidney Disease

Lupus nephritis (LN) is a common complication of systemic lupus erythematosus (SLE). Clinically, approximately 50% of patients may have LN complication. The pathogenesis of LN is mainly related to inflammatory cell infiltration, activation of blood coagulation factor, and the release of inflammatory mediators caused by the deposition of immune complexes in the glomeruli. Patients with renal interstitial disease and vascular disease usually experience much more severe kidney injury and poor prognosis.

Previously, the inflammatory mediator IL-18 was assumed to play an important role in LN. The NLRP3-ASC-caspase-1 signaling pathway has played a crucial part in kidney injury of SLE [79, 80]. The role of P2X7 in the noncanonical pyroptosis pathway has been proven to be related to the activation of caspase-1. In 2013, Zhao et al. [81] found that inhibition of the P2X7/NLRP3/caspase-1 signaling pathway effectively improved LN. A detailed study performed in 2017 confirmed that the inflammatory body NLRP3 was activated in patients and mice with LN, leading to Sertoli cell injury and severe albuminuria [82]. These results suggest the significance of the NLRP3 inflammatory body in LN. Piperine was found to alleviate LN by inhibiting the SLE mouse model and human near-end renal tubular epithelial cell pyroptosis, suggesting a possible role of pyroptosis in the progression of LN [83]. During the occurrence and progression of LN, the relationship between NLRP3 and pyroptosis is not clear. Whether NLRP3 cleaves SGDMD, a specific substrate for pyroptosis, also remains to be studied.

IgA nephropathy (IgAN) is the most frequently seen primary glomerular disease, which means mainly IgA or IgA deposition at the mesangial region of glomeruli, and is accompanied by the presence or absence of other IgAs. It is estimated that 20%–40% patient with IgAN will develop end-stage kidney diseases in 20 years [84]. Therefore, an investigation into the pathogenesis and progressive factors of IgA kidney disease may provide important information for disease treatment.

A study performed 1997 reported the participation of the inflammatory medium IL-1 in the progression of IgAN, suggesting the important role of inflammation in IgAN [85]. It was recently reported that the expression of NLRP3 was significantly increased in kidney tubules of patients with IgAN [86, 87].

### 4.5. Other

At present, the occurrence and progression of many kidney diseases are related to pyroptosis. However, as it is not well studied, this has not been confirmed. Calcium oxalate (CaOx) nephropathy is caused mainly by the excessive accumulation of CaOx in the kidney, eventually leading to end-stage kidney disease. It was found that CaOx crystals could initiate IL-1*β*-dependent innate immunity by activating the NLRP3/ASC/caspase-1 signaling pathway of renal mononuclear phagocytes, causing mouse renal tubular injury and promoting the progression of CaOx nephropathy [88, 89]. Similarly, NLRP3 accelerated glomerular sclerosis in a CaOx nephropathy mouse model and led to progressive renal function failure by mediating an inflammatory reaction [90]. Meanwhile, the long coding RNA LINC00339 was proven to induce renal tubular epithelial cell pyroptosis in patients with CaOx nephropathy by activating NLRP3 via miR-22-3p [91]. Multiple complications can occur in the early stage following kidney transplantation. Further, it has been shown that kidney transplantation could lead to kidney inflammation and far-end liver injury in rats. The markers related to pyroptosis increased markedly, but the correlation between complications resulting from kidney transplantation and pyroptosis needs further investigation [92]. The pathophysiological mechanisms of HIV and HIV-related kidney disease and particulate-induced kidney injury are also closely related to pyroptosis [93, 94]. These findings may provide theoretical rationale for the discovery of new therapeutic targets and the physiopathological alterations in various kidney diseases.

## 5. Potential Medicines for Pyroptosis in Kidney Diseases

Within the studies concerning pyroptosis in the field of kidney diseases, several have focused on proteins related to the pyroptosis signaling pathway, that is, NLRP3, caspase-1, and the inhibitor or activator of GSDMD (Table 2). Quercetin, curcumin, and allopurinol were all found to exert antihyperuricemia and antihyperlipidemic functions and to reduce the release of inflammatory factors by inhibiting the activation of the inflammatory body NLRP3 in renal cells, thereby delaying the progression of DKD [95, 96]. It was also found that CP-456,773, a specific inhibitor of NLRP3, could delay the progression of mouse renal fibrosis owing to its early-stage inhibitory effect [97]. A recent study showed that artemisinin, an anti-inflammatory medicine, could alleviate rat renal tubule interstitial fibrosis by downregulating NLRP3 [98]. Three reagents targeting inflammatory kidney diseases have been found to directly inhibit the activation of NLRP3. Epigallocatechin-3-gallate (EGCG) and Bay11-7082 could largely mitigate the pathological changes of lupus nephropathy, and icariin, a flavonoid from a Chinese herbal medicine, *Herba epimedii*, effectively resists the inflammatory reaction in rats with IgAN [99–101]. In addition, some medicines indirectly inhibit the activation of NLRP3. Mdivi-1, dynamics-related protein 1 (DRP1), alleviated the symptoms of AKI mice by protecting mitochondrial function and inhibiting the activation of NLRP3 [102]. In addition, piperine significantly inhibited the activation of the NLRP3 inflammatory body and reduced the release of proinflammatory cytokines and mouse renal tubule pyroptosis by targeting AMPK to inhibit the development of LN [83]. Zhen-wu-tang is a well-known Chinese herbal medicine formula that inhibits the activation of NLRP3 and delays the reduction of renal function in IgAN rats by enhancing the secretion of renal exosomes [103].

Caspase-1 is a crucial link in the pyroptosis pathway; that is, the inhibitor of caspase-1 also inhibits the occurrence and progression of pyroptosis. Results showed that downregulation of *β*-hydroxybutyrate and expression of proinflammatory cytokines could alleviate pathological injury in AKI mice by inhibiting pyroptosis [104]. Mizoribine could also inhibit the inflammatory reaction in the kidney of renal fibrosis rats by inhibiting caspase-1 and alleviating the symptoms of hypertension [105].

As a specific substrate of pyroptosis, GSDMD is a key target for the regulation of renal pyroptosis. It was found that sodium butyrate alleviated the pyroptosis of glomerular vascular endothelial cells in DKD mice, providing a new target for the treatment of DKD [69]. Recent studies reported that Catalpol and Geniposide could alleviate the symptoms of DKD mice by inhibiting pyroptosis-related proteins, such as GSDMD and GSDMD-N. Of course, there are many potential medicines for kidney diseases by resisting pyroptosis [107, 108]. Riboflavin is considered an anti-inflammatory vitamin because of its antioxidant activity. Riboflavin was found to delay pyroptosis and the release of IL-1*β* and IL-18 by inhibiting the activation of AIM2 and other inflammasomes. At present, studies concerning AIM2 inhibitors and pyroptosis are limited to cancer. Whether AIM2 inhibitors could function by inhibiting pyroptosis requires further investigation. Thus, more research on the topic is highly encouraged [108]. Overall, the present studies have their own limitations and require further confirmation.

## 6. Prospects and Summary

In summary, pyroptosis is mainly regulated by the caspase-1-mediated canonical pathway and the caspase-4/5/11-mediated noncanonical pathway. The occurrence and progression of kidney diseases are more or less related to pyroptosis, and the inflammatory body NLRP3 is the most well-studied. This review examines the significance of pyroptosis in the pathogenesis of acute kidney injury, diabetic kidney, renal fibrosis, and inflammatory kidney disease. The role of pyroptosis as a key target for the treatment of kidney diseases is also highlighted. However, many problems remain to be addressed in the body of research.

At present, the study of pyroptosis is still in its infancy; there are many unanswered questions. For example, although the caspase protein family plays a crucial role in pyroptosis, irrespective of the canonical and noncanonical pathways, they are also the key proteins regulating the middle and late stages of apoptosis. There, when is the relationship between pyroptosis and apoptosis? Is pyroptosis an independent mode of cell death, or is it accompanied by other modes of cell death? Inflammation is a key process mediating pyroptosis, and oxidative stress is a frequently observed factor in inflammatory reactions. Can oxidative stress directly mediate pyroptosis? Studies on pyroptosis have concentrated mainly on two marker proteins, GSDMD and GSDME. Many studies have tended to expose the NT terminals of these two proteins and investigated oligomer-triggered pyroptosis. However, no study has ever reported free NT terminal-mediated pyroptosis. Such questions still need to be proven by a great deal of studies.

Pyroptosis is actually a double-edged sword. When pyroptosis occurs in normal cells of kidney tissues, that is, Sertoli cells and renal tubular epithelial cells, many kidney-related diseases will occur. A majority of studies related to pyroptosis and kidney diseases have focused on this aspect. Meanwhile, moderate pyroptosis is an important immune response of the body that plays very important roles in resisting infection and endogenous risk factors. For example, will removal of injured cells or fibroblasts in kidney tissues through pyroptosis alleviate the degree of kidney-related diseases? Few studies have ever addressed this issue. Therefore, a further study is required to confirm this assumption.

At present, many medicines have been found to function by regulating the pyroptosis pathway. However, studies have largely focused on the field of cancer treatment. The research and development of medicines for the treatment of kidney-related diseases are ongoing. In the future, the medicines targeting at pyroptosis for the treatment of other disease can be tested on kidney-related diseases, and novel medicines for the treatment of kidney-related disease can be designed to target pyroptosis. This will provide a much better direction for the development of medication for kidney-related diseases.

## Figures and Tables

**Table 1:** Morphological and biochemical characteristics of pyroptosis, apoptosis, and necrosis [11–14].

Characteristics	Pyroptosis	Necrosis	Apoptosis
Morphological changes	Cell volume increases; organelles become tumid; cell membrane becomes slightly tumid; many vesicular protuberance are formed. Plasma membrane is damaged and becomes tabular. Before plasma rupture, apoptotic body-like protuberances are formed, called pyroptotic bodies. Inflammatory factors are released.	Cells become significantly tumid; plasma membrane shows explosive ruptures.	Intracellular aggregation of cytoplasm and chromatin, DNA damage, and formation of apoptotic bodies; plasma membrane remain intact.
Caspase family	Caspase-1, 4, 5, 11		Caspase-2, 3, 6, 7, 8, 9, 10
Executor of pore formation	Oligomers are formed at the N-terminal of gasdermin-D and translocated to the plasma membrane to form a nonion selective channel.	Oligomerization of mixed lineage kinase domain-like protein (MLKL) and initiation of plasma membrane translocation; formation of ion-selective channel.	
Test method	Positive TUNEL test is observed, but with a lower intensity than during apoptosis. Positive annexin V staining. Observed directly via electron microscopy.	Dual positive staining for annexin V-PI staining; diffusive DNA electrophoresis bands.	Positive TUNEL test; positive annexin V staining. Directly observed via flow cytometry.
Activation of ADP-ribose polymerase (PARP)	No		Yes

**Table 2:** Potential medicines for the treatment of pyroptosis and kidney diseases.

Type of drugs	Small molecules or drugs	Molecular weight	Molecular formula	Variety of disease	References
NLRP3 inhibitor	Mdivi-1	353.2	C_15_H_10_Cl_2_N_2_O_2_S	AKI	
	Quercetin	302.23	C_15_H_10_O_7_	DKD	[95]
	Curcumin	368.4	C_21_H_20_O_6_	DKD	[96]
	Allopurinol	136.11	C_5_H_4_N_4_O	DKD	[95]
	CP-456,773	404.5	C_20_H_24_N_2_O_5_S	Renal fibrosis	[97]
	Artemisinin	282.33	C_15_H_22_O_5_	Renal fibrosis	[98]
	EGCG	458.4	C_22_H_18_O_11_	LN	[99]
	Bay11-7082	207.25	C_10_H_9_NO_2_S	LN	[100]
	Piperine	285.34	C_17_H_19_NO_3_	LN	[83]
	Icariin	676.7	C_33_H_40_O_15_	IgAN	[101]
Caspase-1 inhibitor	*β*-Hydroxybutyrate	251.28	C_13_H_17_NO_4_	AKI	[104]
	Mizoribine	259.22	C_9_H_13_N_3_O_6_	Renal fibrosis	[105]
GSDMD inhibitor	Sodium butyrate	110.09	C_4_H_7_NaO_2_	DKD	[69]
	Catalpol	362.33	C_15_H_22_O_10_	DKD	[106]
	Geniposide	388.4	C_17_H_24_O_10_	DKD	[107]

## Data Availability

The data used to support the finding of this study are available from the corresponding author upon request.

## Abbreviations

AIM2: Absence of melanoma 2
AKI: Acute kidney injury
AMPK: Adenosine 5′-monophosphate- (AMP-) activated protein kinase
ASC: Apoptosis-associated speck-like protein containing a CARD
ATP: Adenosine triphosphate
bBox: Zinc finger domain
CaOx: Calcium oxalate
CARD: Caspase activation and recruitment domain
CC: A coiled coil domain
CHOP: C/EBP homologous protein
CKD: Chronic kidney disease
DAMPs: Damage-associated molecular patterns
DN: Diabetic nephropathy
DNA: Deoxyribonucleic acid
DRP1: Dynamics-related protein 1
EGCG: Epigallocatechin-3-gallate
ESRD: End-stage renal disease
GSDMD: Gasdermin D
GSDME: Gasdermin E
HIV: Human immunodeficiency virus
HMGB1: High-mobility group box 1 protein
IgAN: IgA nephropathy
IL-1*β*: Interleukin-1*β*
IL-18: Interleukin-18
IRI: Ischemia reperfusion injury
LN: Lupus nephritis
lncRNAs: Long noncoding RNAs
LPS: Lipopolysaccharide
LRRs: Leucine-rich repeats
LT: Anthrax lethal toxin
MALAT1: Metastasis-associated lung adenocarcinoma transcript 1
miR: MicroRNA
MLKL: Mixed lineage kinase domain-like protein
NAIP: NLR family, apoptosis inhibitory protein
NCCD: Nomenclature Committee on Cell Death
NEAT1: lncRNA-Neat 1
NLR: Nucleotide-binding domain, leucine-rich repeat-containing
NLRC4: NLR family, CARD-containing 4
NLRP1: NLR family, PYRIN domain-containing 1
NLRP3: NLR family, PYRIN domain-containing 3
NLRP6: NLR family, PYRIN domain-containing 6
NLRP9: NLR family, PYRIN domain-containing 9
NOD/NACHT: Nucleotide-binding oligomerization domain
P2X7: Purinergic receptor P2X
PAMPs: Pathogen-associated molecular patterns
PARP: ADP-ribose polymerase
PI: Propidium iodide
PYD: Pyrin domain
PYHIN: PYRIN and HIN-200 domain-containing
RNA: Ribonucleic acid
SLE: Systemic lupus erythematosus
T3SS: Bacterial type III secretion system
TLR-4: Toll-like receptor 4
TUNEL: TdT-mediated dUTP nick-end labeling
UUO: Unilateral ureteral occlusion.
